# Expression of SLC26A9 in Airways and Its Potential Role in Asthma

**DOI:** 10.3390/ijms23062998

**Published:** 2022-03-10

**Authors:** Jiraporn Ousingsawat, Raquel Centeio, Rainer Schreiber, Karl Kunzelmann

**Affiliations:** Physiological Institute, University of Regensburg, University Street 31, D-93053 Regensburg, Germany; jiraporn.ousingsawat@vkl.uni-regensburg.de (J.O.); raquel.martins-centeio@vkl.uni-regensburg.de (R.C.); rainer.schreiber@vkl.uni-regensburg.de (R.S.)

**Keywords:** SLC26A9, asthma, cystic fibrosis, airways, Cl^−^ secretion, exocytosis, IL-13

## Abstract

SLC26A9 is an epithelial anion transporter with a poorly defined function in airways. It is assumed to contribute to airway chloride secretion and airway surface hydration. However, immunohistochemistry showing precise localization of SLC26A9 in airways is missing. Some studies report localization near tight junctions, which is difficult to reconcile with a chloride secretory function of SLC26A9. We therefore performed immunocytochemistry of SLC26A9 in sections of human and porcine lungs. Obvious apical localization of SLC26A9 was detected in human and porcine superficial airway epithelia, whereas submucosal glands did not express SLC26A9. The anion transporter was located exclusively in ciliated epithelial cells. Highly differentiated BCi-NS1 human airway epithelial cells grown on permeable supports also expressed SLC26A9 in the apical membrane of ciliated epithelial cells. BCi-NS1 cells expressed the major Cl^−^ transporting proteins CFTR, TMEM16A and SLC26A9 in about equal proportions and produced short-circuit currents activated by increases in intracellular cAMP or Ca^2+^. Both CFTR and SLC26A9 contribute to basal chloride currents in non-stimulated BCi-NS1 airway epithelia, with CFTR being the dominating Cl^−^ conductance. In wtCFTR-expressing CFBE human airway epithelial cells, SLC26A9 was partially located in the plasma membrane, whereas CFBE cells expressing F508del-CFTR showed exclusive cytosolic localization of SLC26A9. Membrane localization of SLC26A9 and basal chloride currents were augmented by interleukin 13 in wild-type CFTR-expressing cells, but not in cells expressing the most common disease-causing mutant F508del-CFTR. The data suggest an upregulation of SLC26A9-dependent chloride secretion in asthma, but not in the presence of F508del-CFTR.

## 1. Introduction

The epithelial anion transporter SLC26A9 (solute carrier 26 family member A9) possibly contributes to airway surface hydration by operating as an uncoupled Cl^−^ transporter [1,2]. SLC26A9, together with the cystic fibrosis transmembrane conductance regulator (CFTR) and the Ca^2+^ activated Cl^−^ channel TMEM16A, is among the most important secretory Cl^−^ pathways in airways. SLC26A9 was suggested to have a role in inflammatory airway diseases, such as cystic fibrosis (CF) and asthma [3,4,5]. SLC26A9 belongs to a family of 10 paralogous mammalian proteins that play a role in tissue-specific ion transport, except of prestin, which mediates electromotility in inner ear outer hair cells [6,7,8,9]. Although operating as a chloride transporter rather than a channel, SLC26A9 nevertheless allows for high Cl^−^ transport rates and substantial anion currents [1]. Not entirely solved is the question of whether SLC26A9 is also able to transport bicarbonate and whether SLC26A9 or CFTR is in charge of basal anion transport in airways [1,10,11,12,13,14,15,16]. The aim of the present study was therefore to examine the contribution of SLC26A9 to airway chloride secretion.

In order to fulfil a chloride secretory function in airways, localization of SLC26A9 in the apical membrane is required. Data on the expression of TMEM16A in human airways are still scarce. Surprisingly, immunofluorescence stainings in well-differentiated airway epithelial cells revealed expression of SLC26A9 near tight junctions [17]. A recent study in human airways also reported expression of SLC26A9 close to tight junctions [18]. However, tight junctional location of SLC26A9 is difficult to reconcile with a Cl^−^ secretory function. Moreover, studies in renal and gastrointestinal mucosa suggested an apical membrane location of SLC26A9 [13,19,20]. Here we present a detailed analysis of the immunolocalization of SLC26A9 in normal, CF and asthmatic airways, as well as porcine airways from wild-type piglets (CFTR+/+) and from piglets lacking expression of CFTR (CFTR−/−). Apical membrane expression in ciliated epithelial cells is also demonstrated in highly differentiated BCi-NS1 human airway epithelial cells [21], which suggests a contribution of SLC26A9 to basal airway transport. BCi-NS1 airway epithelial cells form a tight epithelium typical for a native human airway epithelium. Membrane localization of SLC26A9 was enhanced in wtCFTR-expressing CFBE airway epithelial cells by the interleukin IL-13, which corresponds to earlier data suggesting upregulation of SLC26A9 function in mouse airways by IL-13 and possibly in asthma [5]. The present results support a role of SLC26A9 for airway chloride secretion in asthma, which may not be detectable in airways of CF-patients carrying the F508del-CFTR allele.

## 2. Results

### 2.1. Apical Expression of SLC26A9 in Human Superficial Airway Epithelium Which Is Absent in CF

**Figure 1:**
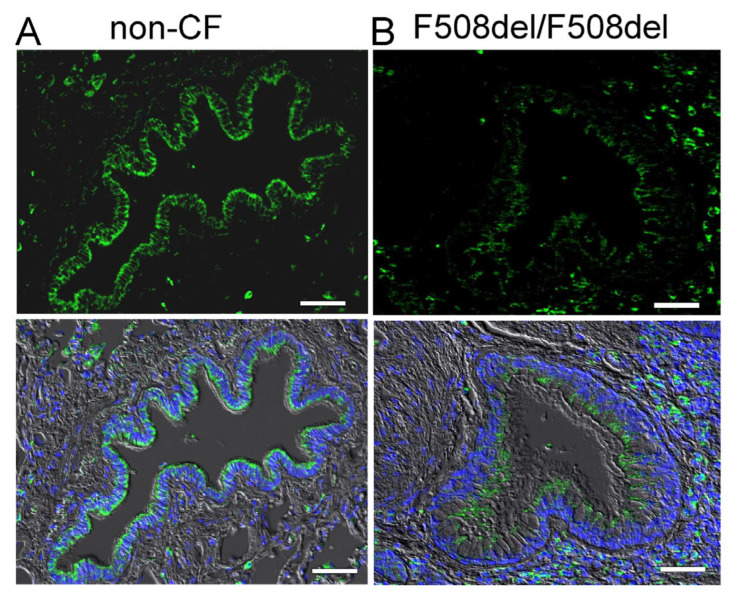
Apical expression of SLC26A9 in human non-CF but not CF superficial airway epithelium. Immunohistochemistry of SLC26A9 (green fluorescence) expressed in non-CF ((**A**) wt-CFTR/wt-CFTR) and CF ((**B**) F508del-CFTR/F508del-CFTR) superficial airway epithelium. Reduced expression and intracellular localization of SLC26A9 is found in CF lungs. Bar = 50 µm. Nuclei were stained by DAPI.

We analysed expression of SLC26A9 in human airways. Immunohistochemistry of SLC26A9 (green fluorescence) shows expression in the apical pole of the superficial epithelium in normal non-CF airways (Figure 1A). In contrast, airways from a CF-patient homozygous for the most common mutation, F508del-CFTR, show a lack of apical expression of SLC26A9 (Figure 1B).

**Figure 2:**
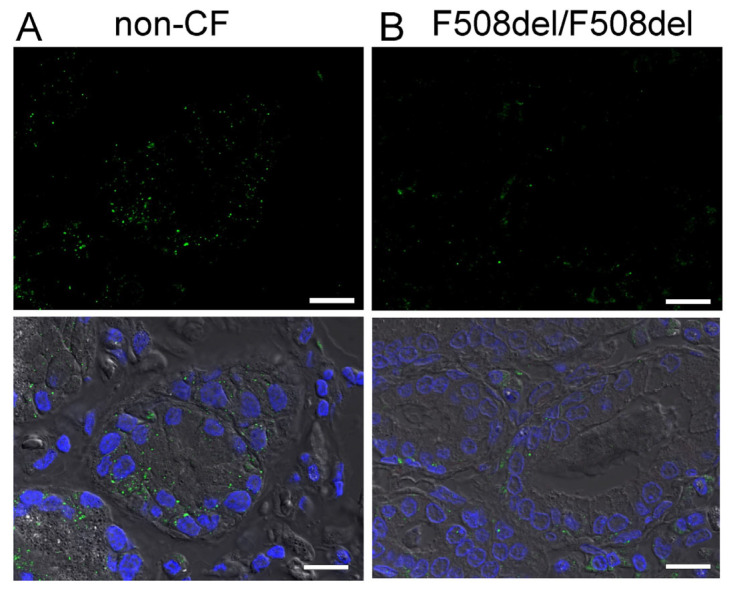
SLC26A9 is not expressed in human airway submucosal glands. Immunohistochemistry of SLC26A9 (green fluorescence) in non-CF (**A**) and CF (**B**) airway submucosal glands. No expression of SLC26A9 is detected in submucosal glands of either non-CF or CF lungs. Bar = 50 µm. Nuclei were stained by DAPI.

Low levels of SLC26A9 are detected in the cytosol of CF-airways. This corresponds to several earlier studies indicating a lack of proper biosynthesis and apical expression of SLC26A9 in cultured CF airway epithelial cells [14,22,23,24]. In contrast to the superficial epithelium, expression of SLC26A9 was not detected in epithelial cells of submucosal glands in either non-CF or CF lungs (Figure 2).

**Figure 3:**
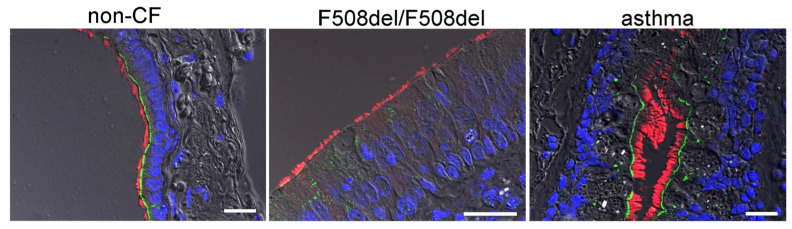
SLC26A9 is located in the apical membrane of ciliated airway epithelial cells. Immunocytochemistry of SLC26A9 (green) and acetylated tubulin (red) in superficial airway epithelium from a non-CF patient, a CF patient (F508del-CFTR/F508del-CFTR) and an asthma patient. SLC26A9 is exclusively localized in the apical membrane of ciliated airway epithelial cells in non-CF and asthmatic lungs but not in CF-lungs from patients carrying the F508del-CFTR/F508del-CFTR mutation. Bar = 50 µm. Nuclei were stained by DAPI.

A more detailed analysis indicates expression of SLC26A9 in the apical membrane of ciliated epithelial cells. Thus, SLC26A9 is coexpressed together with CFTR in ciliated cells, whereas expression of SLC26A9 was not detected in non-ciliated cells (Figure 3). Notably, airways of asthma patients showed normal apical localization of SLC26A9, which even appeared somewhat stronger in some images, when compared to non-asthmatic airways. In contrast, the airways of CF patients homozygous for F508del-CFTR did not show expression of SLC26A9 (Figure 3).

### 2.2. SLC26A9 Is Located in the Apical Membrane of Ciliated Airway Epithelial Cells of CFTR+/+ and CFTR−/− Piglet Lungs

**Figure 4:**
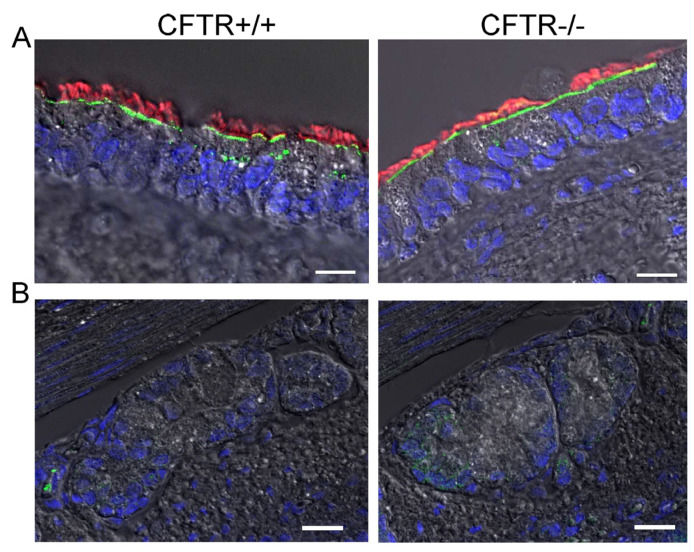
SLC26A9 is located in the apical membrane of ciliated airway epithelial cells of CFTR+/+ and CFTR−/− piglet lungs. (**A**) Immunocytochemistry of SLC26A9 (green) and acetylated tubulin (red) in superficial airway epithelium of wt (CFTR+/+) and CFTR-knockout (CFTR−/−) piglets. (**B**) Expression of SLC26A9 was not detected in airway submucosal glands of CFTR+/+ and CFTR−/− piglets. Bar = 20 µm. Nuclei were stained by DAPI.

In order to provide further evidence for an apical localization of SLC26A9 in airways, we analysed the expression of SLC26A9 in the porcine airway epithelium. To this end, we performed co-staining of SLC26A9 and acetylated tubulin in the superficial airway epithelium of wt-piglets (CFTR+/+) and littermate CFTR-knockout piglets (CFTR−/−). Identically to human airways, superficial airways of CFTR+/+ piglets also demonstrated clear apical expression of SLC26A9 in ciliated epithelial cells (Figure 4A). Remarkably, piglet airways lacking expression of CFTR also expressed SLC26A9 in the apical membrane, indicating that the absence of CFTR does not affect expression of SLC26A9. This is in sharp contrast to F508del-CFTR, which essentially abrogates SLC26A9 expression, as shown above. Like in human airways, expression SLC26A9 is not detected in airway submucosal glands of CFTR+/+ and CFTR−/− piglets (Figure 4B).

**Figure 5:**
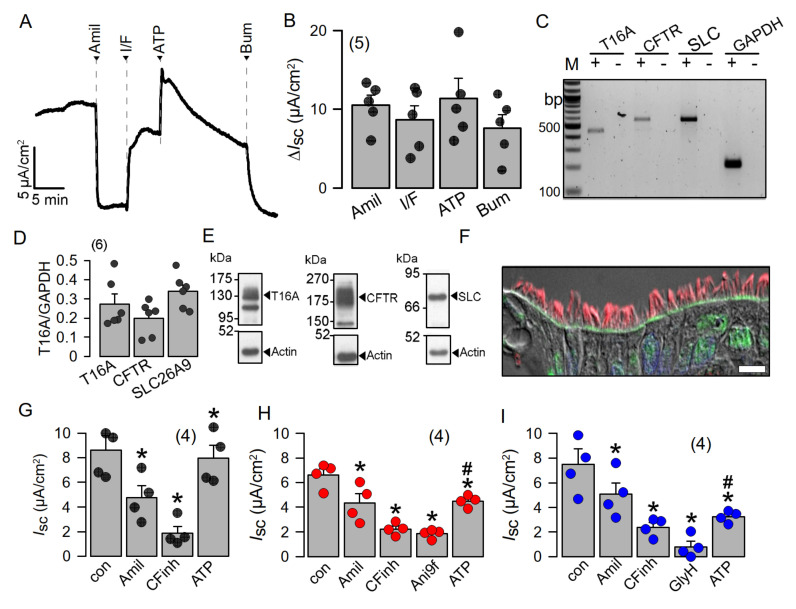
Highly differentiated BCi-NS1 human airway epithelial cells express SLC26A9 in the apical membrane. (**A**,**B**) Using chamber short-circuit current (Isc) recording from highly differentiated BCi-NS1 human airway epithelial cells. Isc is inhibited by amiloride (10 µM), indicating the expression of epithelial Na^+^ channels (ENaC). IBMX (100 µM) and forskolin (2 µM) activate a non-transient CFTR-dependent Isc, which is inhibited by basolateral bumetanide (20 µM). Luminal ATP (100 µM) activates a transient Isc. (**C**,**D**) Expression of the major Cl^−^ secretory proteins, TMEM16A (T16A), CFTR and SLC26A9 (SLC), as detected by RT-PCR. (**E**) Expression of TMEM16A, CFTR and SLC26A9 detected by Western blotting. Blots were performed in duplicate. (**F**) Immunocytochemistry of SLC26A9 (green) and acetylated tubulin (red) in ciliated cells of highly differentiated BCi-NS1 cells grown on permeable supports. Bar = 10 µm. Nuclei were stained by DAPI. (**G**–**I**) Summaries of basal short-circuit currents in BCi-NS1 monolayers, indicating CFTR activity contributing to basal Isc, as detected by CFTRinh172 (CFinh; 10 µM). The TMEM16A-inhibitor Ani9f (10 µM) largely reduces ATP-activated TMEM16A-dependent Cl^−^ secretion. The inhibitor GlyH101 (Gly; 10 µM) further inhibits basal Isc after inhibition of CFTR by CFTRinh172. Mean ± SEM (number of tissues). * Significant effects by compounds (*p* < 0.05; paired *t*-test). # Significant difference compared to G (*p* < 0.05; unpaired *t*-test).

Highly differentiated BCi-NS1 human airway epithelial cells express SLC26A9 in the apical membrane, which may contribute to basal Cl^−^ conductance. To further examine the role of SLC26A9 in human airway epithelial cells, we made use of the BCi-NS1 basal cell line which maintains multipotent differentiation capacity [21]. When grown on permeable supports, this cell line forms a tight airway epithelium with transport properties essentially identical to those of the original human airway epithelium. The use of chamber recordings demonstrated a basal Na^+^ absorption, which is inhibited by amiloride, the inhibitor of epithelial Na^+^ channels (ENaC). Steady-state and transient short-circuit currents (Isc) were activated by an increase in intracellular cAMP (using IBMX and forskolin) and by the purinergic agonist ATP, respectively (Figure 5A,B). Activated Cl^−^ secretion was inhibited by the NKCC1 inhibitor bumetanide. Polarized BCi-NS1 epithelia express similar amounts of the major Cl^−^ channels and transporters CFTR, TMEM16A and SLC26A9, as demonstrated by semiquantitative RT-PCR and Western blotting (Figure 5C–E). For TMEM16A, the bands for the glycosylated (130 kDa) and non-glycosylated (100 kDa) protein are shown. CFTR is detected as a so-called band c (180 kDa), and a band b (150 kDa). SLC26A9 shows a band at 90 kDa. The uncropped blots show a number of additional lower-molecular-weight bands for SLC26A9, which are known to be caused by several splice versions. SLC26A9 was also nicely detected in this cell line in the apical membrane of ciliated epithelial cells (Figure 5F). After inhibition of Na^+^ absorption by amiloride, we found that the CFTR inhibitor CFTRinh172 substantially decreased constitutive (basal) Isc, although the epithelium was not previously stimulated by IBMX/forskolin. A transient Isc was activated by stimulation with ATP (Figure 5G). Additional inhibition of TMEM16A by Ani9f [25] did not affect constitutive Isc, but inhibited ATP-activated Isc, suggesting that TMEM16A does not contribute to basal Cl^−^ secretion in human airways (Figure 5H). GlyH101 (GlyH) was reported to inhibit SLC26A9 [14]. We applied GlyH after inhibition of CFTR with CFTRinh172 and found additional inhibition of Isc that could well be due to inhibition of SLC26A9 (Figure 5I). Thus, both CFTR and SLC26A9 may contribute to basal constitutive Cl^−^ secretion in human airways.

### 2.3. IL-13 Augments Membrane Expression of SLC26A9 and Induces Basal Cl^−^ Currents in CFBE Airway Epithelial Cells Expressing wtCFTR, but Not in Cells Expressing F508del-CFTR

**Figure 6:**
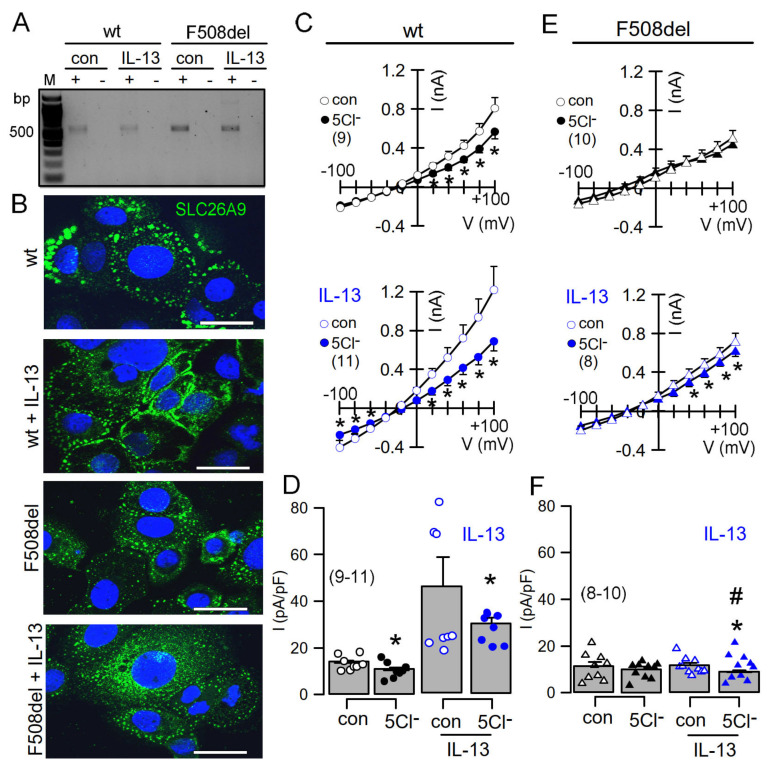
IL-13 augments membrane expression of SLC26A9 and induces basal currents in CFBE airway epithelial cells expressing wtCFTR but not in cells expressing F508del-CFTR. (**A**) Expression of SLC26A9 detected by RT-PCR in CFBE airway epithelial cells stably expressing wtCFTR (wt) or F508delCFTR (F508del). Cells were treated with IL-13 (20 ng/72 h), which did not enhance expression of SLC26A9. (**B**) Immunohistochemistry of SLC26A9 in CFBE/wtCFTR and CFBE/F508delCFTR cells. Exposure of the cells to IL-13 enhanced membrane expression of endogenous SLC26A9 in cells expressing wtCFTR, but not in cells expressing F508del-CFTR. Note that in CFBE/wtCFTR cells, some SLC26A9 is located in the plasma membrane even in the absence of IL-13, whereas CFBE/F508del cells show cytoplasmic expression of SLC26A9. Bars = 20 µm. (**C**–**F**) Current/voltage relationships showing basal currents in CFBE/wtCFTR (**C**,**D**) and CFBE/F508delCFTR (**E**,**F**) cells in the absence or presence of IL-13, as well as the effect of removal of chloride (5Cl^−^) from the extracellular bath solution. Basal Cl^−^ transport detected by 5Cl^−^ replacement was larger in CFBE/wtCFTR cells when compared to CFBE/F508del cells. Mean ± SEM (number of cells). * Significant inhibition by 5Cl^−^ (*p* < 0.05; paired *t*-test). # Significant difference compared to wtCFTR (*p* < 0.05; unpaired *t*-test).

We made use of the human airway epithelial cell line CFBE stably expressing wtCFTR or F508del-CFTR in order to further analyse CFTR-dependent regulation of SLC26A9. SLC26A9 might be upregulated in asthmatic airways [5]. It was proposed that SLC26A9-mediated Cl^−^ secretion prevents mucus obstruction in airways of IL-13 treated mice. Here, we stimulated CFBE cells with IL-13, which did not upregulate relatively low levels of SLC26A9 expressed in wt and F508del cells (Figure 6A). However, exposure of the cells to IL-13 enhanced membrane expression of endogenous SLC26A9 in CFBE cells expressing wtCFTR (Figure 6B). In contrast, F508del-CFTR-expressing cells showed exclusive intracellular localization of SLC26A9, and IL-13 was unable to translocate SLC26A9 to the plasma membrane. We analysed the corresponding whole-cell currents using patch clamp and found a constitutive Cl^−^ current that could be inhibited by removal of extracellular Cl^−^ (5Cl^−^). Moreover, exposure to IL-13 further enhanced constitutive whole-cell currents and augmented 5Cl^−^-induced current inhibition (Figure 6C,D). Unlike wtCFTR-expressing cells, F508del-CFTR-expressing cells did not present constitutive currents and showed only a tiny constitutive Cl^−^ transport after incubation with IL-13 (Figure 6E,F). Taken together, the present data clearly indicate apical membrane expression of SLC26A9 in human and porcine airways, which is fully reproduced in polarized BCi-NS1 human airway epithelia. Both CFTR and SLC26A9 may contribute to constitutive chloride secretion, but CFTR clearly dominates. The Th-2 cytokine IL-13 enhances membrane expression of SLC26A9, thereby increasing constitutive Cl^−^ secretion.

## 3. Discussion

### 3.1. SLC26A9 Is Expressed in the Apical Membrane of the Airway Epithelium

The present data clearly show expression of SLC26A9 in the apical membrane of airways taken from original lung sections. During the course of this study, it became clear that airway epithelial cells require complete polarization and differentiation in order to express SLC26A9 apically. Thus, plastic-grown airway epithelial cells, such as BCi-NS1 or CFBE, show very little membrane expression. In additional experiments, we tried to biotinylate SLC26A9 in membranes of non-polarized (plastic-grown) BCi-NS1 and CFBE cells but found very little SLC26A9 in the biotinylated fraction (data not shown). Moreover, SLC26A9 expressed in HEK293 cells shows almost exclusively cytosolic expression [24], similar to a stably SLC26A9-expressing FRT cell line generated recently [26]. Thus, proper analysis of the function of SLC26A9 in airways requires a polarized environment.

Localization of SLC26A9 near tight junctions was reported previously [17,18]. Although this was not a regular finding in the present study, we occasionally observed a lateral staining of SLC26A9 in native airways (not shown). We speculate that the airway epithelium in these rare lung sections was not fully differentiated, possibly due to inflammation. Importantly, SLC26A9 was only found in ciliated epithelial cells, which corresponds well to the interaction of SLC26A9 and other SLC26A solute transporters with CFTR, as described in several previous reports [9,14,27,28,29,30].

### 3.2. Plasma Membrane Expression of SLC26A9 Is CFTR-Dependent and Is Augmented by IL-13

Expression of SLC26A9 was essentially absent in human F508del-CFTR/F508del-CFTR airways. Moreover, SLC26A9 present in non-polarized CFBE cells expressing F508del-CFTR was detected exclusively in the cytosol. Both findings highlight the pronounced abrogating effect of F508del-CFTR on SLC26A9 [22,23]. In contrast, SLC26A9 staining was exclusively apical in non-CF lungs and was even found in the membrane of non-polarized CFBE cells expressing wtCFTR. Notably, airways of CFTR knockout piglets demonstrated normal SLC26A9 expression in the apical plasma membrane, suggesting that SLC26A9 is able to traffic to its physiological location in the absence of CFTR, whereas coexpression with F508del-CFTR abrogates biosynthesis and trafficking of SLC26A9.

**Figure 7:**
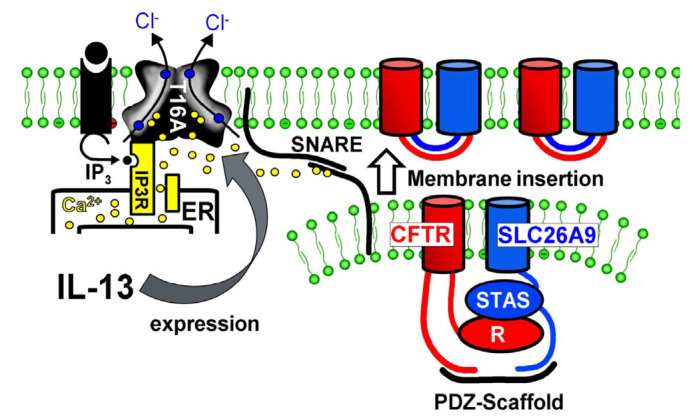
Enhanced membrane expression of SLC26A9 in asthma. Proposed mechanism for enhanced SLC26A9 expression in airways exposed to IL-13. Exposure to IL-13 leads to enhanced expression of TMEM16A. TMEM16A expressed in the apical membrane tethers the endoplasmic reticulum (ER) to the apical membrane and leads to enhanced compartmentalized Ca^2+^ signalling. Enhanced intracellular apical Ca^2+^ concentrations facilitate membrane insertion of CFTR and SLC26A9 via the Ca^2+^-regulated SNARE (soluble ***N***-ethylmaleimide-sensitive factor attachment protein receptor) complex.

It was found that IL-13 treatment increases Cl^−^ secretion in airways of wild-type but not Slc26a9-deficient mice. Thus, an upregulation of SLC26A9-dependent Cl^−^ secretion may help to prevent mucus obstruction in asthma [5]. In airways of asthma patients, we found normal expression of SLC26A9, and in some sections, SLC26A9 staining appeared somewhat enhanced (not shown). When we treated CFBE/wtCFTR cells with IL-13, we also found an increase in SLC26A9 membrane expression, whereas SLC26A9-mRNA expression was not enhanced. Enhanced membrane expression was paralleled by enhanced constitutive Cl^−^ conductance, suggesting enhanced SLC26A9 conductance in asthmatic airways. It should be mentioned that IL-13 (and IL-4) also strongly upregulated airway expression of the Ca^2+^-activated Cl^−^ channel TMEM16A in vivo and in vitro [31]. It was shown earlier that TMEM16A is essential for exocytosis and proper membrane insertion [32,33,34,35]. This is demonstrated most impressively in patients lacking expression of functional TMEM16A, as well as in TMEM16A-knockout mice [32,33,34]. Thus, Th-2 cytokines, such as IL-4 and IL-13, augment membrane expression of both CFTR and SLC26A9 because SLC26A9 and CFTR physically interact via the STAS/R domain and scaffold proteins containing PDZ domains (Figure 7). Such a mechanism provides enhanced constitutive Cl^−^ and fluid secretion and antagonizes mucus plugging, as proposed earlier [5,33,34,35].

A limitation of the present study is the limited number of available lung sections. Thus, subsequent studies should analyse expression of SLC26A9 in CF lungs from patients with different class 1–7 mutations, as well as the effect of CFTR correctors on correcting the plasma membrane location of SLC26A9. Moreover, quantitative comparison of SLC26A9 membrane expression in normal and asthmatic airways would be helpful to clarify whether asthmatic airways express more apical SLC26A9. Nevertheless, the results suggest a limited contribution of SLC26A9 to airway Cl^−^ secretion. This may therefore suggest an additional role of SLC26A9, maybe for bicarbonate transport. Plasma membrane location of SLC26A9 was essentially unchanged in lungs from CFTR-knockout piglets. This may explain why we did not find evidence for acidification of the airway surface liquid (ASL) in lungs of CFTR-knockout piglets in our previous study [36]. Finally, the present results support a protective function of SLC26A9 in asthmatic lungs and attach a beneficial role to IL-13, which is found to be enhanced in asthma.

## 4. Materials and Methods

### 4.1. Tissues, Cell Culture and Treatment

Airway sections from CFTR+/+ and CFTR−/− littermate piglets were generously provided by Prof. Dr. Nick Klymiuk (Molekulare Tierzucht und Biotechnologie, LMU Munich [37]. All animal procedures were performed according to the German Animal Welfare Act with permission of the local regulatory authority of LMU Munich. Piglets were euthanized within the first 24 h after birth under Ketamine (Ursotamin^®^, Serumwerk Bernburg, Bernburg Germany) and Azaperone (Stresnil^®^, Elanco Animal Health, Bad Homburg, Germany) anaesthesia by intracardiac injection of T61^®^ (Intervet, Unterschleissheim, Germany). At least three human and piglet samples (wtCFTR, F508del-CFTR, CFTR+/+, CFTR−/−) were analysed. The human lung sections were kindly provided by Prof. Eric Verbeken and Prof. Kris De Boeck (University of Leuven, B-3000 Leuven, Belgium).

Human cystic fibrosis bronchial epithelial cell lines (CFBE) stably expressing wtCFTR or F508del-CFTR [38] were a generous gift from Dr. J.P. Clancy (University of Alabama at Birmingham, Birmingham, AL, USA). CFBE wtCFTR and F508del cells were cultured in MEM media supplemented with 2 mM L-glutamine and 2.5 μg/mL puromycin. Generation of CFBE cells stably expressing mCherry-Flag-wtCFTR and F508del-CFTR were described in a previous report [39] and were a generous gift from Prof. Dr. M.D. Amaral (University of Lisbon, Portugal). CFBE mCherry-Flag-CFTR cells were cultured in high-glucose DMEM supplemented with 2 mM L-glutamine, 1 mM pyruvate, 10 μg/mL blasticidin and 2 μg/mL puromycin. All media were supplemented with 10% heat-inactivated foetal calf serum (all culture media and supplements were from Capricorn Scientific, Ebsdorfergrund, Germany). BCi-NS1 cells (kindly provided by Prof. R. Crystal, Weill Cornell Medical College, New York, NY, USA) [21] were cultured in supplemented bronchial epithelial cell growth medium (BEGM; Lonza, Basel, Switzerland). For polarization, BCi-NS1 cells were seeded onto permeable supports for up to 30 days (Snapwell #3801, Corning, New York, USA) in an air–liquid (ALI) interface. All cells were cultured at 37 °C in a humidified atmosphere of 5% (*v*/*v*) CO_2_. For the IL-13 experiment, cells were treated with 20 ng/mL IL-13 (Enzo Life Sciences, Lörrach, Germany) for 72 h in OptiMEM reduced serum medium (Gibco/Thermo Fisher Scientific, Waltham, MA, USA); IL-13 was refreshed every day. Cell membrane surface biotinylation was performed as described earlier [31]. SLC26A9 was detected by Western blotting using rabbit polyclonal anti-SLC26A9 (custom SCH-DRA; Davids, #P125) and HRP-conjugated goat anti-rabbit antibody.

### 4.2. RT-PCR

For RT-PCR, total RNA from BCi-NS1 and CFBE human airway epithelial cells was isolated using NucleoSpin RNA II columns (Macherey-Nagel, Düren, Germany). Total RNA (0.5 µg/25 µL reaction) was reverse-transcribed using random primer (Promega, Mannheim, Germany) and M-MLV reverse transcriptase RNase H minus (Promega, Mannheim, Germany). Each RT-PCR reaction contained sense (0.5 µM) and antisense primer (0.5 µM) (Table 1), 0.5 µL cDNA and GoTaq polymerase (Promega, Mannheim, Germany). After 2 min at 95 °C, cDNA was amplified (targets of 35 cycles, reference GAPDH 25 cycles) for 30 s at 95 °C, 30 s at 56 °C and 1 min at 72 °C. PCR products were visualized by loading on Midori Green Xtra (Nippon Genetics Europe) containing agarose.

### 4.3. Western Blot

Protein was isolated from filter-grown BCi-NS1 cells using a lysis buffer containing 25 mM Tris-HCl pH 7.4, 150 mM NaCl, 1 mM EDTA, 5% glycerol, 0.43% Nonidet P-40, 100 mM dithiothreitol (both from PanReac AppliChem, Barcelona, Spain) and 1× protease inhibitor mixture (Roche, Basel, Switzerland). Proteins were then separated by 8.5% SDS-PAGE and transferred to a PVDF membrane (GE Healthcare, Munich, Germany). Membranes were incubated overnight at 4 °C with rabbit primary antibodies against TMEM16A (#ab64085, Abcam, Cambridge, UK) and α-SLC26A9 (Davids Biotechnologie, Regensburg, Germany) and a mouse primary antibody against CFTR (#596, Cystic Fibrosis Foundation Therapeutics, Bethesda, MA, USA). Rabbit anti-actin (#A2066; Sigma-Aldrich, St. Louis, MO, USA) was used as a loading control. Membranes were then incubated with horseradish peroxidase (HRP)-conjugated goat anti-rabbit/sheep anti-mouse secondary antibodies at room temperature for 2 h, and immunoreactive signals were visualized using a SuperSignal HRP chemiluminescence substrate-detection kit (#34577; Thermo Fisher Scientific, Waltham, MA, USA).

### 4.4. Immunocytochemistry

For staining, cells were fixed for 10 min with methanol and acetone (5:1) mixing solution at −20 °C. Paraffin-embedded lung sections and monolayer CFBE sections (5 µm) were deparaffinized with xylene and rehydrated through a series of ethanol. The standard protocol for immunofluorescence was performed. In brief, after blocking with 5% bovine serum albumin in PBS, sections or cells were incubated in rabbit anti-SCL26A9 antibody (1:100, raised against mouse SCL26A9 aa 11–29, DRAAYSLSLFDDEFEKKDR, used in porcine tissues) or rabbit anti-SCL26A9 antibody (1:100, Novus Biologicals, Wiesbaden Nordenstadt, Germany, used in human tissues/cells) overnight at 4 °C. Antigen retrieval was performed by Davids Biotechnologie, Regensburg, Germany in pre-heated Tris-EDTA buffer (pH 9.0) for 15 min, using a microwave before blocking (Figure S1). Cells were incubated with secondary anti-rabbit antibody conjugated with Alexa Fluor 488 or Alexa Fluor 546 (1:400) for 1h at room temperature. Cilia were stained using a monoclonal antibody against acetylated tubulin produced in mouse (T7451, Sigma-Aldrich, Taufkirchen, Germany). Nuclei were counterstained with DAPI. Airway sections or cells were mounted with fluorescence mounting medium (DAKO Cytomation, Hamburg, Germany). Immunofluorescence was examined with an Axio Observer microscope equipped with Axiocams 503 mono, ApoTome.2 and ZEN 3.0 (blue edition) software (Zeiss, Oberkochen, Germany).

*Transepithelial Using chamber recordings:* BCi-NS1 cells polarized on permeable supports were measured under short-circuit conditions in non-perfused chambers with bicarbonate-Ringer solution (mmol/L: NaCl 118.75; KH_2_PO_4_ 0.4; K_2_HPO_4_ 1.6; Glucose 5; MgSO_4_ 1; Ca-Gluconate 1.3, NaHCO_3_ 25; bubbled with 95% O_2_/5% CO_2_). Comprehensive methods have been detailed in previous reports [36,40].

*Patch Clamp:* Cells were patch-clamped when grown on coated glass coverslips, and experiments were performed at 37 °C. Patch pipettes were filled with a cytosolic-like solution containing (in mM): KCl 30, K-Gluconate 95, NaH_2_PO_4_ 1.2, Na_2_HPO_4_ 4.8, EGTA 1, Ca-Gluconate 0.758, MgCl_2_ 1.03, D-Glucose 5, ATP 3; pH 7.2. The intracellular (pipette) Ca^2+^ activity was 0.1 µM. The bath was perfused continuously at a rate of 4–6 mL/min with Ringer’s solution (in mM): NaCl 145, KH_2_PO_4_ 0.4, K_2_HPO_4_ 1.6, Glucose 5, MgCl_2_ 1, Ca-Gluconate 1.3. The substitution of extracellular Cl^−^ (5 mM) was replaced by gluconate. Patch pipettes had an input resistance of 2–5 MΩ, and whole-cell currents were corrected for serial resistance. The current–voltage (I–V) relationship was determined by pulsing from the holding potential of −100 mV to test potentials between −100 and +100 mV increasing in 20 mV increments. Currents were recorded using an EPC-9 computer-controlled amplifier and PULSE software (HEKA, Lambrecht, Germany), as well as Chart software (AD Instruments, Spechbach, Germany).

## Figures and Tables

**Table 1:** PCR primers.

Gene Accession Number	Primer	Size (bp)
CFTR NM_000492.4	s: 5′- CTCATTAGAAGGAGATGCTCCTG as: 5’- GCTCTTGTGGACAGTAATATATCG	568
SLC26A9 NM_052934.4	s: 5´- CATTTGCTGTGCGCTTTCTG as: 5´- CCGCTTCTCCTGCTTCTTG	568
TMEM16A NM_018043.7	s: 5´- CGACTACGTGTACATTTTCCG as: 5´- GATTCCGATGTCTTTGGCTC	445
GAPDH NM_001289726	s: 5´- GTATTGGGCGCCTGGTCAC as: 5´- CTCCTGGAAGATGGTGATGG	200

## Data Availability

MDPI Research Data Policies at https://www.mdpi.com/ethics.

## Supplementary Materials

The following supporting information can be downloaded at: https://www.mdpi.com/article/10.3390/ijms23062998/s1.

## References

[1] Alper S.L., Sharma A.K. (2013). The SLC26 gene family of anion transporters and channels. Mol. Aspects Med..

[2] Amlal H., Xu J., Barone S., Zahedi K., Soleimani M. (2013). The chloride channel/transporter Slc26a9 regulates the systemic arterial pressure and renal chloride excretion. J. Mol. Med..

[3] Anagnostopoulou P., Riederer B., Duerr J., Michel S., Binia A., Agrawal R., Liu X., Kalitzki K., Xiao F., Chen M. (2012). SLC26A9-mediated chloride secretion prevents mucus obstruction in airway inflammation. J. Clin. Investig..

[4] Bebok Z., Collawn J.F., Wakefield J., Parker W., Li Y., Varga K., Sorscher E.J., Clancy J.P. (2005). Failure of cAMP agonists to activate rescued deltaF508 CFTR in CFBE41o- airway epithelial monolayers. J. Physiol..

[5] Benedetto R., Centeio R., Ousingsawat J., Schreiber R., Janda M., Kunzelmann K. (2020). Transport properties in CFTR−/− knockout piglets suggest normal airway surface liquid pH and enhanced amiloride-sensitive Na(+) absorption. Pflugers Arch..

[6] Benedetto R., Ousingsawat J., Cabrita I., Pinto M., Lerias J., Wanitchakool P., Schreiber R., Kunzelmann K. (2019). Plasma membrane localized TMEM16 Proteins are Indispensable for expression of CFTR. J. Mol. Med..

[7] Benedetto R., Ousingsawat J., Wanitchakool P., Zhang Y., Holtzman M.J., Amaral M., Rock J.R., Schreiber R., Kunzelmann K. (2017). Epithelial Chloride Transport by CFTR Requires TMEM16A. Sci. Rep..

[8] Bertrand C.A., Mitra S., Mishra S.K., Wang X., Zhao Y., Pilewski J.M., Madden D.R., Frizzell R.A. (2017). The CFTR trafficking mutation F508del inhibits the constitutive activity of SLC26A9. Am. J. Physiol. Lung Cell Mol. Physiol..

[9] Bertrand C.A., Zhang R., Pilewski J.M., Frizzell R.A. (2009). SLC26A9 is a constitutively active, CFTR-regulated anion conductance in human bronchial epithelia. J. Gen. Physiol..

[10] Botelho H.M., Uliyakina I., Awatade N.T., Proenca M.C., Tischer C., Sirianant L., Kunzelmann K., Pepperkok R., Amaral M.D. (2015). Protein traffic disorders: An effective high-throughput fluorescence microscopy pipeline for drug discovery. Sci. Rep..

[11] Cabrita I., Benedetto R., Wanitchakool P., Lerias J., Centeio R., Ousingsawat J., Schreiber R., Kunzelmann K. (2020). TMEM16A Mediated Mucus Production in Human Airway Epithelial Cells. Am. J. Respir. Cell Mol. Biol..

[12] Centeio R., Ousingsawat J., Schreiber R., Kunzelmann K. (2021). CLCA1 Regulates Airway Mucus Production and Ion Secretion Through TMEM16A. Int. J. Mol. Sci..

[13] Chang M.H., Plata C., Zandi-Nejad K., Sindic A., Sussman C.R., Mercado A., Broumand V., Raghuram V., Mount D.B., Romero M.F. (2009). Slc26a9-Anion Exchanger, Channel and Na(+) Transporter. J. Membr. Biol..

[14] Dallos P., Fakler B. (2002). Prestin, a new type of motor protein. Nat. Rev. Mol. Cell Biol..

[15] Dorwart M.R., Shcheynikov N., Wang Y., Stippec S., Muallem S. (2007). SLC26A9 is a Cl(-) channel regulated by the WNK kinases. J. Physiol..

[16] El Khouri E., Toure A. (2014). Functional interaction of the cystic fibrosis transmembrane conductance regulator with members of the SLC26 family of anion transporters (SLC26A8 and SLC26A9): Physiological and pathophysiological relevance. Int. J. Biochem. Cell Biol..

[17] Klymiuk N., Mundhenk L., Kraehe K., Wuensch A., Plog S., Emrich D., Langenmayer M.C., Stehr M., Holzinger A., Kroner C. (2012). Sequential targeting of CFTR by BAC vectors generates a novel pig model of cystic fibrosis. J. Mol. Med..

[18] Ko S.B., Shcheynikov N., Choi J.Y., Luo X., Ishibashi K., Thomas P.J., Kim J.Y., Kim K.H., Lee M.G., Naruse S. (2002). A molecular mechanism for aberrant CFTR-dependent HCO(3)(-) transport in cystic fibrosis. EMBO J..

[19] Ko S.B., Zeng W., Dorwart M.R., Luo X., Kim K.H., Millen L., Goto H., Naruse S., Soyombo A., Thomas P.J. (2004). Gating of CFTR by the STAS domain of SLC26 transporters. Nat. Cell Biol..

[20] Lerias J., Pinto M., Benedetto R., Schreiber R., Amaral M., Aureli M., Kunzelmann K. (2018). Compartmentalized crosstalk of CFTR and TMEM16A (ANO1) through EPAC1 and ADCY1. Cell Signal..

[21] Liu X., Li T., Riederer B., Lenzen H., Ludolph L., Yeruva S., Tuo B., Soleimani M., Seidler U. (2015). Loss of Slc26a9 anion transporter alters intestinal electrolyte and HCO3(-) transport and reduces survival in CFTR-deficient mice. Pflugers Arch..

[22] Loriol C., Dulong S., Avella M., Gabillat N., Boulukos K., Borgese F., Ehrenfeld J. (2008). Characterization of SLC26A9, facilitation of Cl(-) transport by bicarbonate. Cell Physiol. Biochem..

[23] Needham P.G., Goeckeler-Fried J.L., Zhang C., Sun Z., Wetzel A.R., Bertrand C.A., Brodsky J.L. (2021). SLC26A9 is selected for endoplasmic reticulum associated degradation (ERAD) via Hsp70-dependent targeting of the soluble STAS domain. Biochem. J..

[24] Ohana E., Yang D., Shcheynikov N., Muallem S. (2008). Diverse transport modes by the Solute Carrier 26 family of anion transporters. J. Physiol..

[25] Ousingsawat J., Schreiber R., Kunzelmann K. (2011). Differential contribution of SLC26A9 to Cl(-) conductance in polarized and non-polarized epithelial cells. J. Cell Physiol..

[26] Park J.H., Ousingsawat J., Cabrita I., Bettels R.E., Große-Onnebrink J., Schmalstieg C., Biskup S., Reunert J., Rust S., Schreiber R. (2020). TMEM16A deficiency: A potentially fatal neonatal disease resulting from impaired chloride currents. J. Med. Genet.

[27] Pereira S.V.N., Ribeiro J.D., Bertuzzo C.S., Marson F.A.L. (2018). Interaction among variants in the SLC gene family (SLC6A14, SLC26A9, SLC11A1, and SLC9A3) and CFTR mutations with clinical markers of cystic fibrosis. Pediatric Pulmonol..

[28] Pinto M.C., Quaresma M.C., Silva I.A.L., Railean V., Ramalho S.S., Amaral M.D. (2021). Synergy in Cystic Fibrosis Therapies: Targeting SLC26A9. Int. J. Mol. Sci..

[29] Quesada R., Dutzler R. (2019). Alternative chloride transport pathways as pharmacological targets for the treatment of cystic fibrosis. J. Cyst. Fibrosis.

[30] Rakonczay Z., Hegyi P., Hasegawa M., Inoue M., You J., Iida A., Ignáth I., Alton E.W., Griesenbach U., Ovári G. (2008). CFTR gene transfer to human cystic fibrosis pancreatic duct cells using a Sendai virus vector. J. Cell Physiol..

[31] Rode B., Dirami T., Bakouh N., Rizk-Rabin M., Norez C., Lhuillier P., Lorès P., Jollivet M., Melin P., Zvetkova I. (2012). The testis anion transporter TAT1 (SLC26A8) physically and functionally interacts with the cystic fibrosis transmembrane conductance regulator channel: A potential role during sperm capacitation. Hum. Mol. Genet..

[32] Salomon J.J., Spahn S., Wang X., Fullekrug J., Bertrand C.A., Mall M.A. (2016). Generation and functional characterization of epithelial cells with stable expression of SLC26A9 Cl- channels. Am. J. Physiol. Lung Cell Mol. Physiol..

[33] Sato Y., Thomas D.Y., Hanrahan J.W. (2019). The anion transporter SLC26A9 localizes to tight junctions and is degraded by the proteasome when co-expressed with F508del-CFTR. J. Biol. Chem..

[34] Seidler U., Nikolovska K. (2019). Slc26 Family of Anion Transporters in the Gastrointestinal Tract: Expression, Function, Regulation, and Role in Disease. Comp. Physiol..

[35] Seo Y., Kim J., Chang J., Kim S.S., Namkung W., Kim I. (2018). Synthesis and biological evaluation of novel Ani9 derivatives as potent and selective ANO1 inhibitors. Eur. J. Med. Chem..

[36] Strug L.J., Gonska T., He G., Keenan K., Ip W., Boelle P.Y., Lin F., Panjwani N., Gong J., Li W. (2016). Cystic fibrosis gene modifier SLC26A9 modulates airway response to CFTR-directed therapeutics. Hum. Mol. Genet..

[37] Walter J.D., Sawicka M., Dutzler R. (2019). Cryo-EM structures and functional characterization of murine Slc26a9 reveal mechanism of uncoupled chloride transport. eLife.

[38] Walters M.S., Gomi K., Ashbridge B., Moore M.A., Arbelaez V., Heldrich J., Ding B.S., Rafii S., Staudt M.R., Crystal R.G. (2013). Generation of a human airway epithelium derived basal cell line with multipotent differentiation capacity. Respir. Res..

[39] Xu J., Henriksnäs J., Barone S., Witte D., Shull G.E., Forte J.G., Holm L., Soleimani M. (2005). SLC26A9 is expressed in gastric surface epithelial cells, mediates Cl-/HCO3- exchange, and is inhibited by NH4+. Am. J. Physiol. Cell Physiol..

[40] Xu J., Song P., Miller M.L., Borgese F., Barone S., Riederer B., Wang Z., Alper S.L., Forte J.G., Shull G.E. (2008). Deletion of the chloride transporter Slc26a9 causes loss of tubulovesicles in parietal cells and impairs acid secretion in the stomach. Proc. Natl. Acad. Sci. USA.

