# Supplementary material for: Expression of SLC26A9 in Airways and Its Potential Role in Asthma
Source: Int J Mol Sci. 2022 Mar 10;23(6):2998. doi: 10.3390/ijms23062998 (PMC8950296; doi:10.3390/ijms23062998)

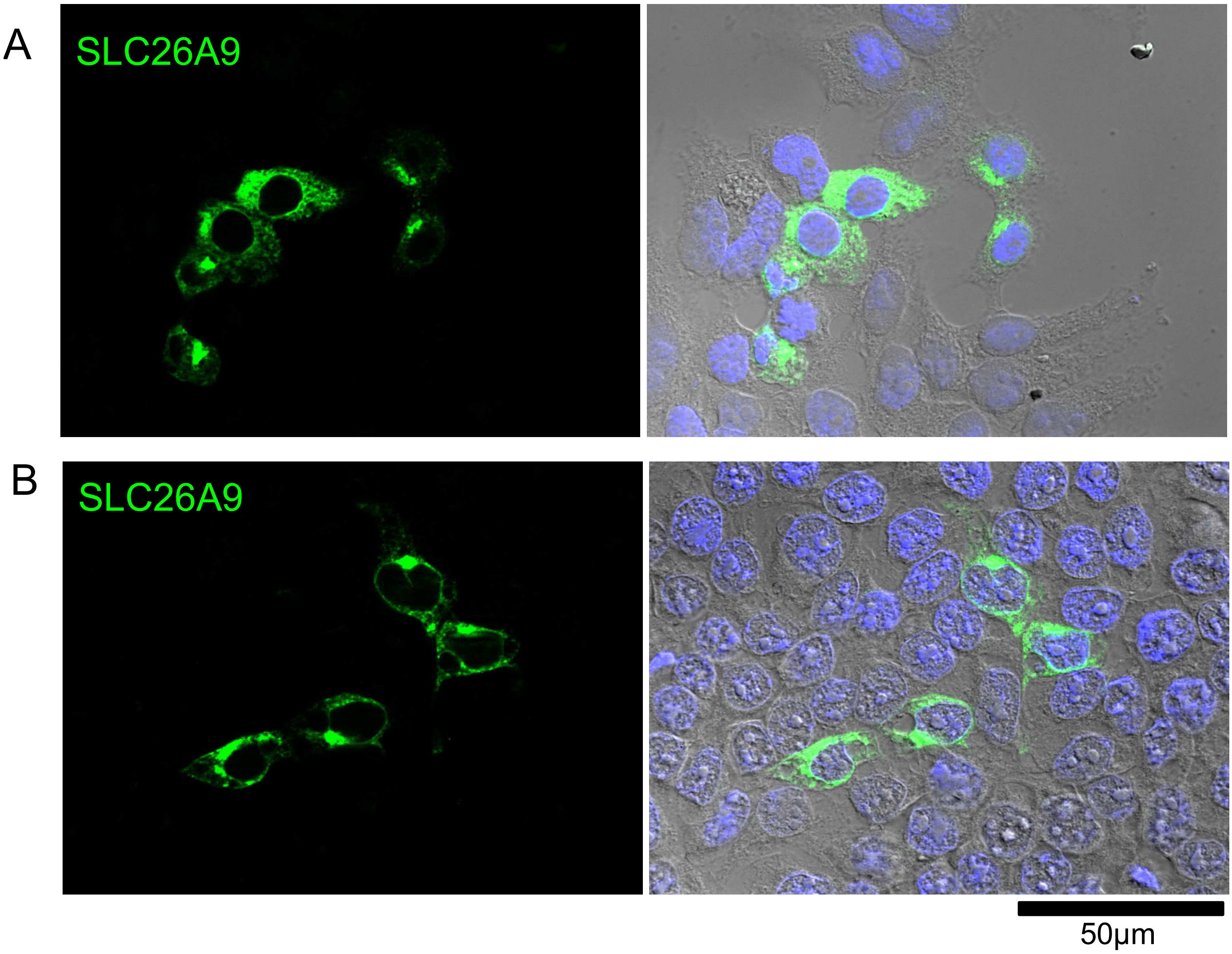

Supplementary Figure 1: *Specificity of immunostaining using SLC26A9 antibodies*. Images show SLC26A9 transfected HEK293 cells as indicated by green fluorescence of SLC26A9 expressing cells. Primary antibodies used were A) custom made AB from Davids Biotechnology, Regensburg, Germany, B) Novus Biologicals, Wiesbaden Nordenstadt, Germany). Non-transfected HEK293 cells, which do not express endogenous SLC26A9, were without fluorescence. Nuclei were staing by DAPI.

Fig. 5C

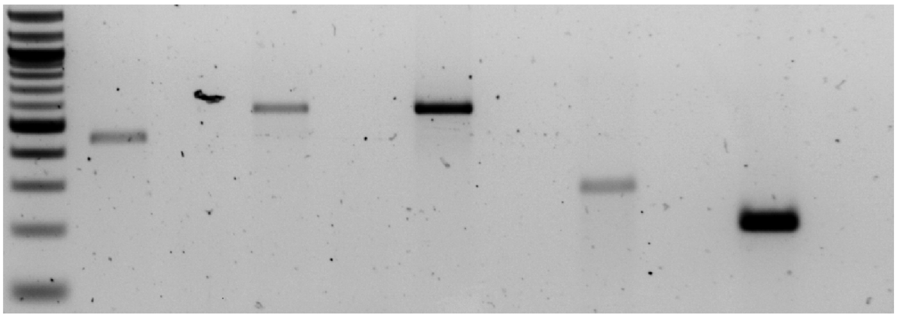

Fig. 5E

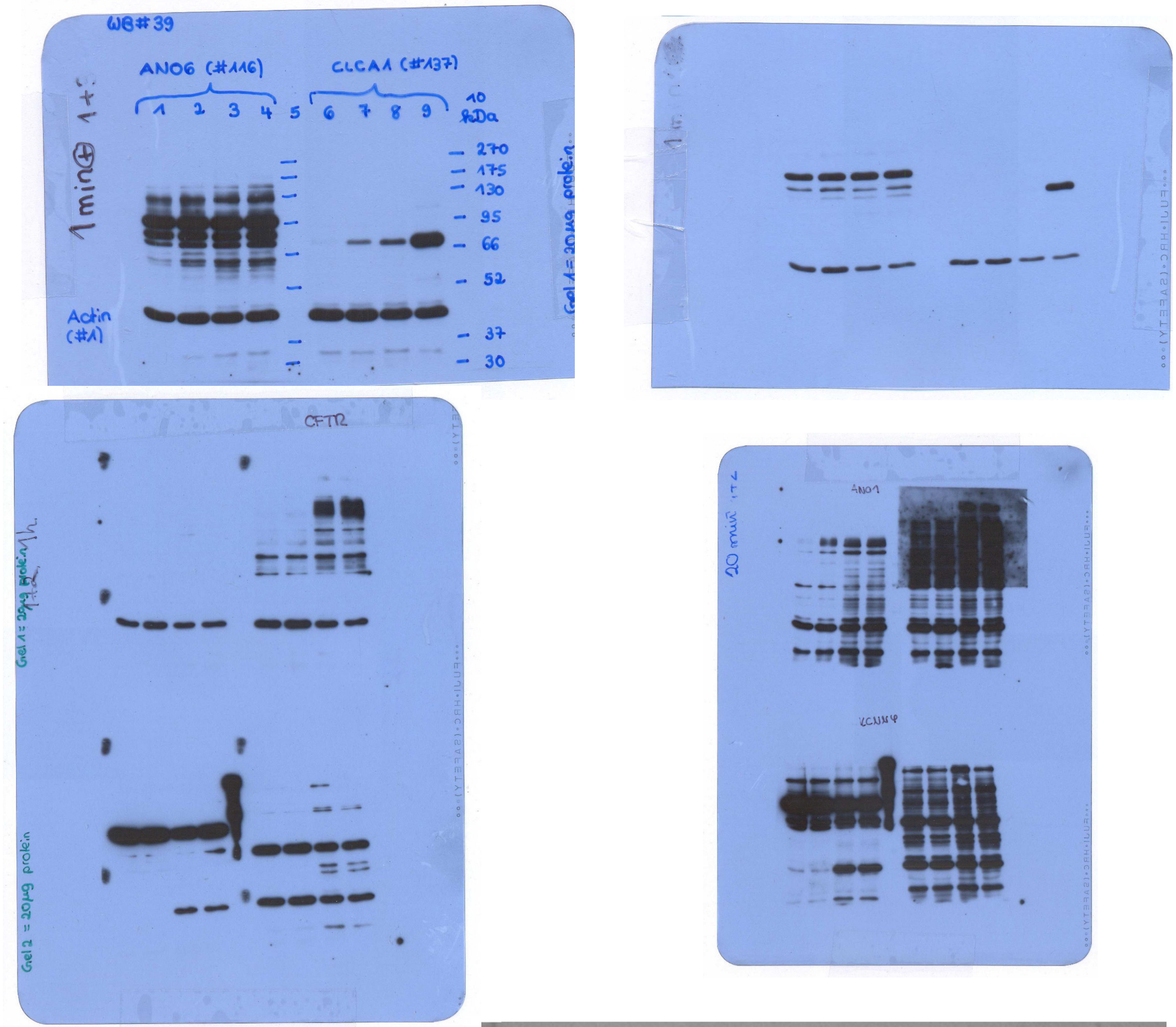

Fig. 6A

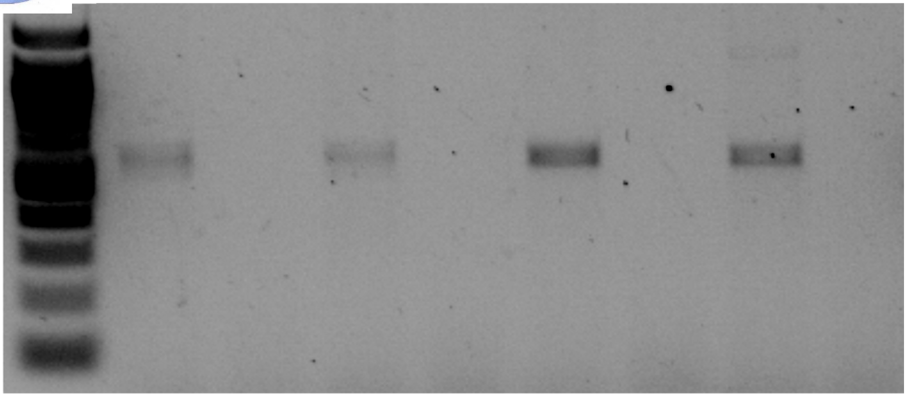

Supplement: Supplementary file 1 [file ijms-23-02998-s001.zip › ijms-1611463-supplementary.pdf]
